# Epigenetic Regulations of Immediate Early Genes Expression Involved in Memory Formation by the Amyloid Precursor Protein of Alzheimer Disease

**DOI:** 10.1371/journal.pone.0099467

**Published:** 2014-06-11

**Authors:** Aurélie Hendrickx, Nathalie Pierrot, Bernadette Tasiaux, Olivier Schakman, Pascal Kienlen-Campard, Charles De Smet, Jean-Noël Octave

**Affiliations:** 1 Alzheimer Dementia, Institute of Neurosciences (IoNS), Université catholique de Louvain, Brussels, Belgium; 2 Cell Physiology, Institute of Neurosciences (IoNS), Université catholique de Louvain, Brussels, Belgium; 3 Genetic & Epigenetic Alterations in Genomes, de Duve Institute, Université catholique de Louvain, Brussels, Belgium; National Center for Geriatrics and Gerontology, Japan

## Abstract

We previously demonstrated that APP epigenetically regulates *Egr1* expression both in cultured neurons and *in vivo*. Since *Egr1* is an immediate early gene involved in memory formation, we wondered whether other early genes involved in memory were regulated by APP and we studied molecular mechanisms involved. By comparing prefrontal (PF) cortex from wild type (APP+/+) and APP knockout mice (APP−/−), we observed that APP down regulates expression of four immediate early genes, *Egr1*, *c-Fos*, *Bdnf* and *Arc*. Down regulation of *Egr1*, *c-Fos* and *Bdnf* transcription resulted from a decreased enrichment of acetylated histone H4 on the corresponding gene promoter. Further characterization of H4 acetylation at *Egr1* and *c-Fos* promoters revealed increased acetylation of H4K5 and H4K12 residues in APP−/− mice. Whereas APP affected *Egr1* promoter activity by reducing access of the CREB transcription factor, its effect on *c-Fos* appeared to depend on increased recruitment of HDAC2 histone deacetylase to the gene promoter. The physiological relevance of the epigenetic regulation of *Egr1* and *c-Fos* gene transcription by APP was further analyzed following exposure of mice to novelty. Although transcription of *Egr1* and *c-Fos* was increased following exposure of APP+/+ mice to novelty, such an induction was not possible in APP−/− mice with a high basal level of expression of these immediate early genes. Altogether, these results demonstrate that APP-mediated regulation of *c-Fos* and *Egr1* by different epigenetic mechanisms is needed for their induction during exposure to novelty.

## Introduction

One of the first steps involved in memory formation is the rapid induction of immediate early genes (IEGs) transcription in the brain. *Egr1* is an IEGs member of early growth response family of zinc fingers transcription factors, widely studied for its role in reconsolidation of memory and its ability to establish long term spatial localization memories [1], [2]. In mouse, *Egr1* is needed for late-phase LTP, is involved in long-term memory formation and controls neuronal function in the hippocampus [3], [4].

Expression of *c-Fos* protein in neurons is induced by a wide range of sensory stimuli [5], [6] and several studies have demonstrated the role of *c-Fos* in the establishment of neuronal plasticity by regulating downstream gene expression [7], [8]. *c-Fos* deficient animals show spatial and associative learning deficits correlated with decrease in synaptic plasticity [9]. Although expressed at low levels in the brain, transcription of *c-Fos* gene sharply increases after exposure to novelty. Both *Egr1* and *c-Fos* mRNA levels are significantly increased 30–45 minutes after exposure to novelty, in particular in the CA1 and CA3 regions of the hippocampus as well as in the PF cortex [10].

Contrary to *Egr1* and *c-Fos*, *Arc* protein is not a transcription factor but an effector synaptic protein involved in multiple neuronal pathways [11]. *Arc* induction occurs in the hippocampus and the cortex following exploration of a novel environment [12]. LTP and synaptic activation also induce *Arc* expression both at the mRNA and protein levels [13].

*Bdnf* is a member of neurotrophin family involved in neuronal growth and survival [14], in the development of dendritic network modulating synaptic transmission [15] and in the regulation of synaptic plasticity and memory formation [16]. In rodents, at least 22 different *Bdnf* mRNA are produced by alternative splicing of a primary transcript, and 9 alternative promoters control *Bdnf* gene transcription, but exon IV promoter is preferentially induced by neuronal activity [17], [18]. Chromatin remodeling also controls *Bdnf* gene transcription in neurons [19].

We previously demonstrated that the amyloid precursor protein (APP) of Alzheimer disease regulates, at the epigenetic level, the transcription of the *Egr1* gene [20]. Induction of IEGs expression is closely related to a final change in chromatin remodeling that allows gene expression [21]. Recruitment of CREB on the Egr1 and c-Fos gene promoters induces an increase in histones acetylation mediated by the CBP/P300 acetyltransferase, and the concomitant depletion of HDACs [19].

Here we show for the first time that APP fosters a low level of *Egr1* and *c-Fos* expression in mouse PF cortex, by inhibiting CREB recruitment and improving HDAC2 recruitment on the corresponding gene promoters. A low constitutive level of *Egr1* and *c-Fos* expression mediated by APP is needed for their induction during exposure of mice to novelty.

## Materials and Methods

### Animals

Five months old mice C57Bl/6J APP+/+ and APP−/− were used in this study. Mice were obtained from The Jackson laboratory and backcrossed for more than five generations in the CD1 genetic background. All animals had access to food *ad libidum* and were housed under controlled temperature (22°C) and with a 12 h light cycle (starting at 7 am). One week before experiments, mice were moved to the experimental platform. As previously described, the behavioral test of exposure to novelty was performed in a square openfield (60×60×40 cm) with gray plastic walls. Mice were able to explore this environment during 15 minutes and sacrificed by CO_2_ inhalation after a resting period of 30 minutes. Another group of mice were directly sacrificed without exposure to the open field. After brain dissection, PF cortex and hippocampus were directly placed in liquid nitrogen, frozen at −80°C or directly crushed and used in further experiments. All manipulations on mice have been approved by the local ethics committee of the catholic University of Louvain and follow the European legislation.

### RNA Extraction and Quantitative Real Time PCR

Total RNA was purified using Trizol method (Tripure, Roche). Reverse transcription (RT) and quantitative (q) real time PCR (q-RT PCR) were performed with the iScript cDNA synthesis Kit and the iQ SYBR Green supermix using a iCycler MyIQ2 multicolor Real-Time PCR detection system (Biorad). The relative amplification of cDNA fragments was calculated by the 2-ΔΔCt method. q-RT PCR primer sequences used were as follows: *Egr1* Forward: TCCTCTCCATCACATGCCTG, *Egr1* Reverse: CACTCTGACACATGCTCCAG, *c-Fos* Forward: GATGTTCTCGGGTTTCAACG, *c-Fos* Reverse: GGAGAAGGAGTCGGCTGG. *GAPDH* Forward: ACCCAGAAGACTGTGGATGG, *GAPDH* Reverse: ACACATTGGGGGTAGGAACA, *Arc* Forward: GCTGAGCTCTGCTCTTCTTCA, *Arc* Reverse: GGTGAGCTGAAGCCACAAAT *Bdnf* Forward: GCGGACCCATGGGACTCT, *Bdnf* Reverse: CTGCTGCTGTAGTGACCGA.

### Chromatin Immunoprecipitation (ChIP)

ChIP was performed using the EZ-ChIP Assay kit (Millipore) according to manufacturer instructions and as described previously [20]. Chromatin was isolated and pooled from the two PF cortices of a single mouse. ChIP experiments were performed using a minimum of 3 mice per group. Chromatin was sheared in an ice bath by a 25 cycles of 30 sec on/off sonication using the “Bioruptor UCD-20” sonicator (Diagenode). Samples were kept on ice during 30 s between two pulses. An aliquot of precleared chromatin was collected as the input. The samples were incubated overnight at 4°C with the antibodies of interest: 5 µg anti-H4Ac (Millipore), 5 µg anti-H3Ac (Millipore), 5 µl anti-H2BAc (Abcam); 10 µl anti-Tip60 (SantaCruz), 5 µl anti-HDAC2 (Abcam), 10 µl anti-CREB total (Millipore), 3 µl of acetylated H4K5, K12, K16 (Active Motif). The immunoprecipitated chromatin was analyzed by quantitative PCR with primers designed to amplify short regions of the promoters of genes of interest.

qPCR primers were as follows: *Egr1* Forward: GTGCCCACCACTCTTGGAT, *Egr1* Reverse: CGAATCGGCCTCTATTTCAA, *c-Fos* Forward: GAAAGCCTGGGGCGTAGAGT, *c-Fos* Reverse: CCTCAGCTGGCGCCTTTAT, *Arc* Forward: CAGCATAAATAGCCGCTGGT, *Arc* Reverse: AGTGTGGCAGGCTCGTC, *Bdnf* exIV Forward: GCGCGGAATTCTGATTCTGGTAAT, *Bdnf* exIV Reverse: GAGAGGGCTCCACGCTGCCTTGACG. *GAPDH* Forward: AGAGAGGGAGGAGGGGAAATG, *GAPDH* Reverse: AACAGGGAGGAGCAGAGAGCAC. The quantification method used is based on the ratio between immunoprecipitated chromatin and input chromatin.

### Protein Analysis

Nuclear extracts of mouse PF cortex were prepared in 0.25 M sucrose buffer (sucrose 0.25 M, Tris 50 mM, EDTA 1 mM, imidazole 3 mM, pH 7.0 + proteases inhibitor cocktail). Samples were centrifuged 10 min at 250 g and nuclear fraction was resuspended in Laemmli buffer. All samples were sonicated before protein assay (BCA Pierce, Thermoscientific) and Western blotting was performed on 20 µg of protein lysates. Membranes were incubated overnight at 4°C with the primary antibodies; anti-HDAC2 1∶1500 (Abcam), anti-H3 1∶10000 (Millipore), anti-tubulin 1∶4000 (Sigma), anti-CREB and phospho-CREB 1∶1000 (Millipore). Washes with PBS-Tween (0.005%) were followed by incubation with secondary antibody (1∶10 000 anti-mouse or anti-rabbit IgG) (GE Healthcare) coupled to horseradish peroxidase and revealed by ECL. For quantification, the membranes were stripped and reincubated with an anti-tubulin or an anti-H3 antibody. Immunoreactive bands were quantified with an electrophoresis Gel Doc 2000 imaging system coupled to a Quantity one software (Bio-Rad).

### Statistical Analysis

All results were expressed as mean ± standard deviation (SD) values. Statistical significance was determined by student’s t-test for two-group comparisons or one-way analysis of variance (ANOVA) followed by Bonferroni’s multiple comparisons test for multi-group comparison.

## Results

### 1. APP Decreases the Transcription of 4 Different IEGs in the Mouse PF Cortex

**Figure 1:**
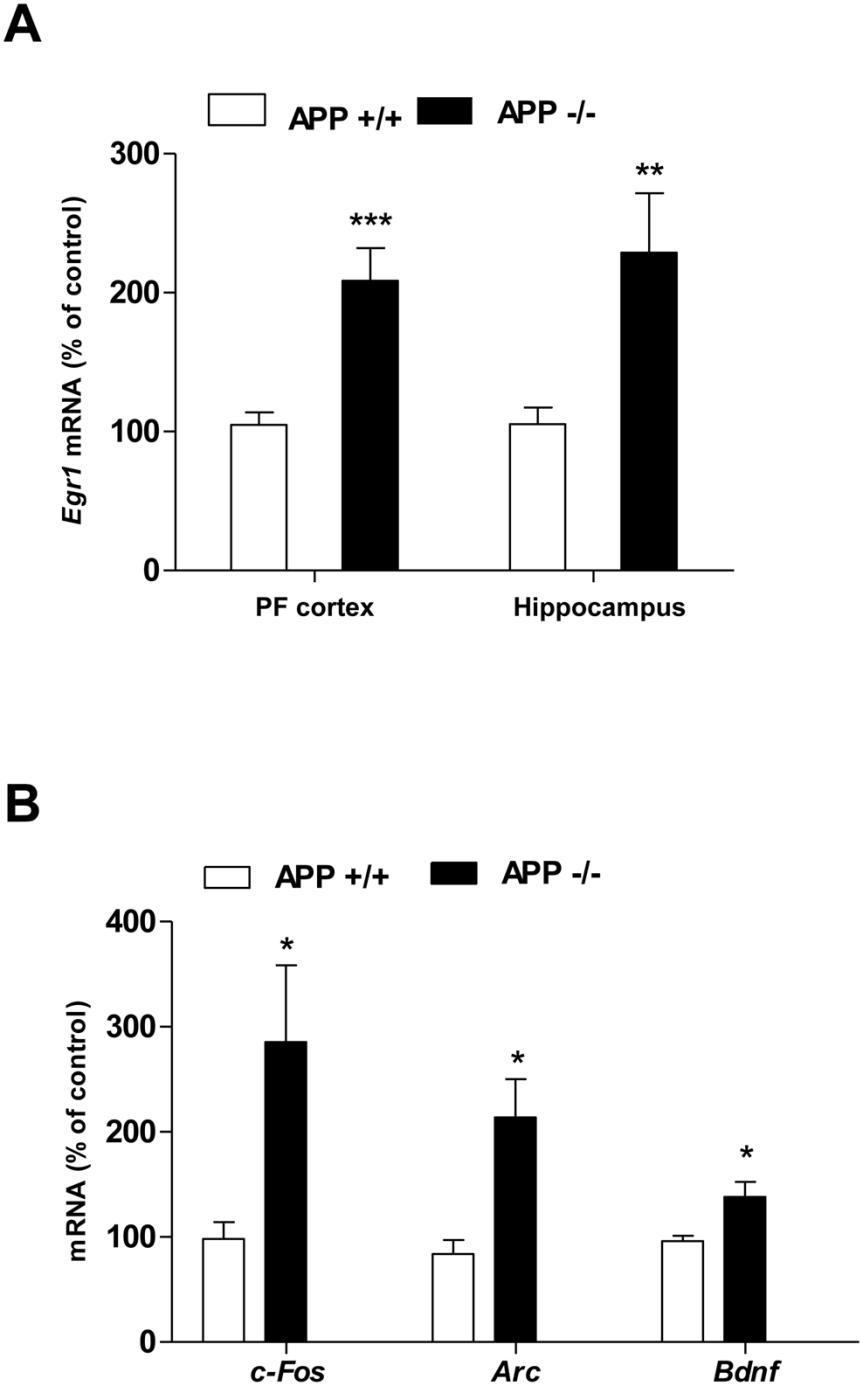
APP regulates IEGs expression in mouse PF cortex. A) *Egr1* mRNA levels were quantified by q-RT PCR in APP+/+ and APP−/− PF cortex (n = 9), and in the hippocampus (n = 6). B) q-RT PCR method was used to quantify mRNA levels of *c-Fos*, *Arc* and *Bdnf* in APP+/+ and APP−/− PF cortex (n = 6). Values were normalized to the *GAPDH* mRNA, and expressed as percentage of APP+/+, mean ± SD. Student’s t-test: ***p<0.001,**p<0.01, *p<0.05.

RNA was prepared from the hippocampus and PF cortex of APP+/+ and APP−/− mice, and qRT-PCR were performed to quantify IEGs mRNAs. Results presented in figure 1A indicate a 2.5 increase in *Egr1* mRNA levels in PF cortex of APP−/− mice, confirming our previous results [20]. We wondered whether transcription of other IEGs involved in memory formation could also be regulated by APP. Results presented figure 1B indicate a significant increase in *c-Fos*, *Arc* and *Bdnf* mRNA levels in APP−/− mice. Levels of IEGs mRNA were normalized to *GAPDH* mRNA levels, which were not regulated by APP, as 3 other housekeeping genes (*Actin, peptidylprolyl isomerase A, and β-glucuronidase*) (Figure S1). In addition, expression of 2 other IEGs (*c-Jun and Homer-1a*) was not affected by APP (Figure S1).

These results therefore suggest that APP is able to regulate transcription of several IEGs involved in memory formation.

### 2. APP Affects Enrichment of Acetylated Histone H4 on Genes Promoters

**Figure 2:**
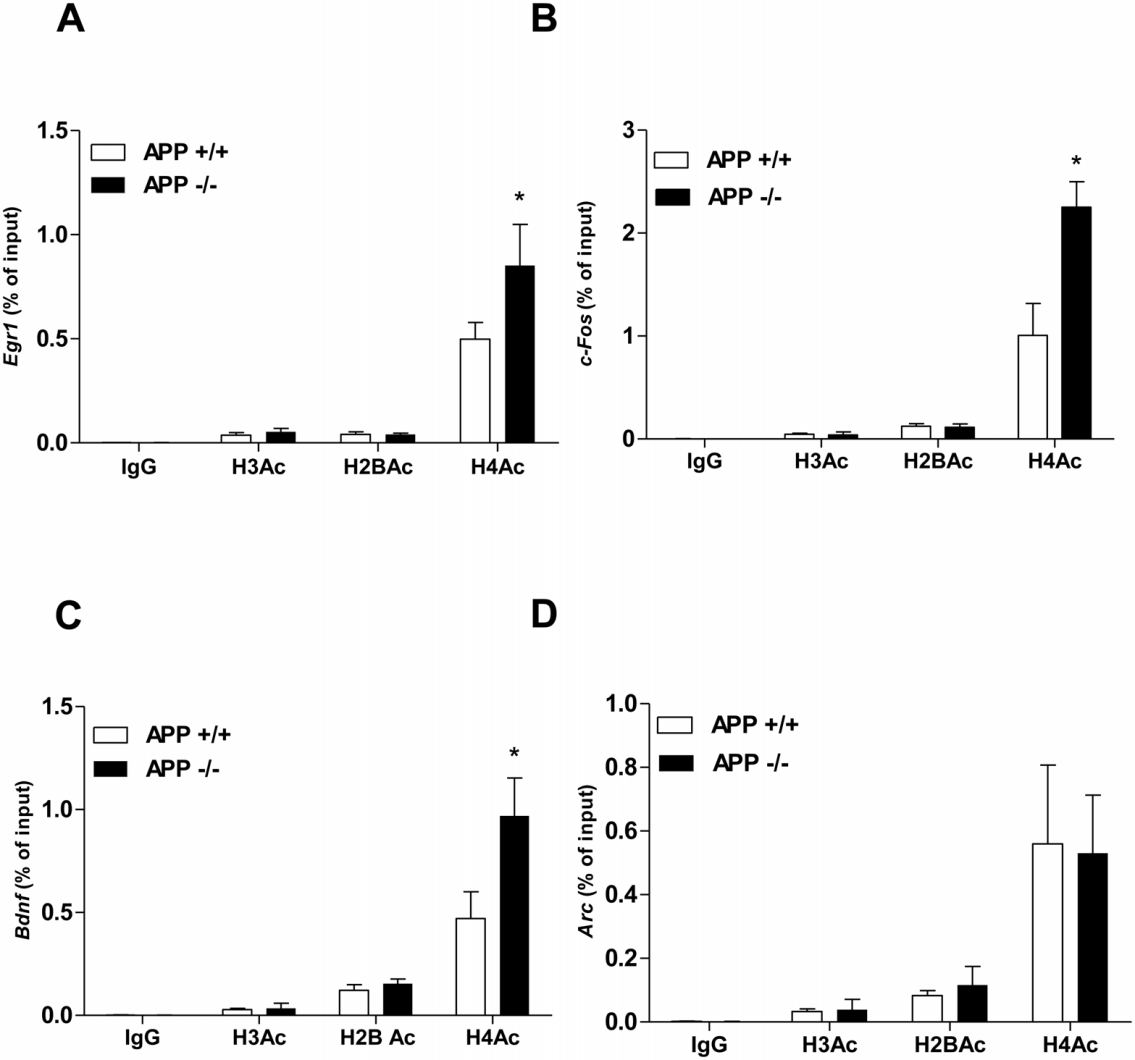
Analysis of histone acetylation by ChIP assays on IEGs promoters. ChIP experiments were performed with chromatin obtained from APP+/+ and APP−/− PF cortex. Immunoprecipitation was completed with antibody recognizing normal mouse IgG as negative control, or anti- H3Ac, H2BAc and H4Ac antibodies. The quantification of immunoprecipitated chromatin and the normalization versus total chromatin (input) was assessed by real-time qPCR with primers designed on A) *Egr1*, B) *c-Fos*, C) *Arc* and D) *Bdnf* exon IV promoters; *p<0.05. All results were obtained from at least 3 mice per group and per antibody, and are expressed as mean ± SD.

We previously observed that the control of *Egr1* gene transcription by APP is independent of the APP intracellular domain (AICD) but occurs at the epigenetic level [20]. Therefore, we wondered whether APP could control transcription of other IEGs by the same mechanism. To that aim, ChIP experiments were performed using anti-acetylated histones antibodies. Results presented in figure 2A clearly demonstrated a specific enrichment of acetylated H4, but not H3 and H2B, on the *Egr1* promoter in APP−/− mice, confirming our previous results [20]. Interestingly, the same specific enrichment of acetylated H4 was measured on both the *c-Fos* (Figure 2B) and *Bdnf* (Figure 2C) gene promoters in APP−/− mice. No modification in H3Ac, H2BAc and H4Ac enrichment on the *GAPDH* gene promoter was observed in APP+/+ and APP−/− experimental conditions (Figure S2). Enrichment of acetylated H4 was not observed on the *Arc* gene promoter in APP−/− mice (Figure 2D), indicating that a different molecular mechanism is involved in the APP-mediated regulation of *Arc* gene expression. This conclusion, together with a much weaker up regulation of *Bdnf* expression in APP−/− mice (Figure 1) led us to focus on the molecular mechanisms involved in the regulation of *Egr1* and *c-Fos* expression by APP.

### 3. APP Specifically Regulates Acetylation of Histone H4 at Lysines 5 and 12

**Figure 3:**
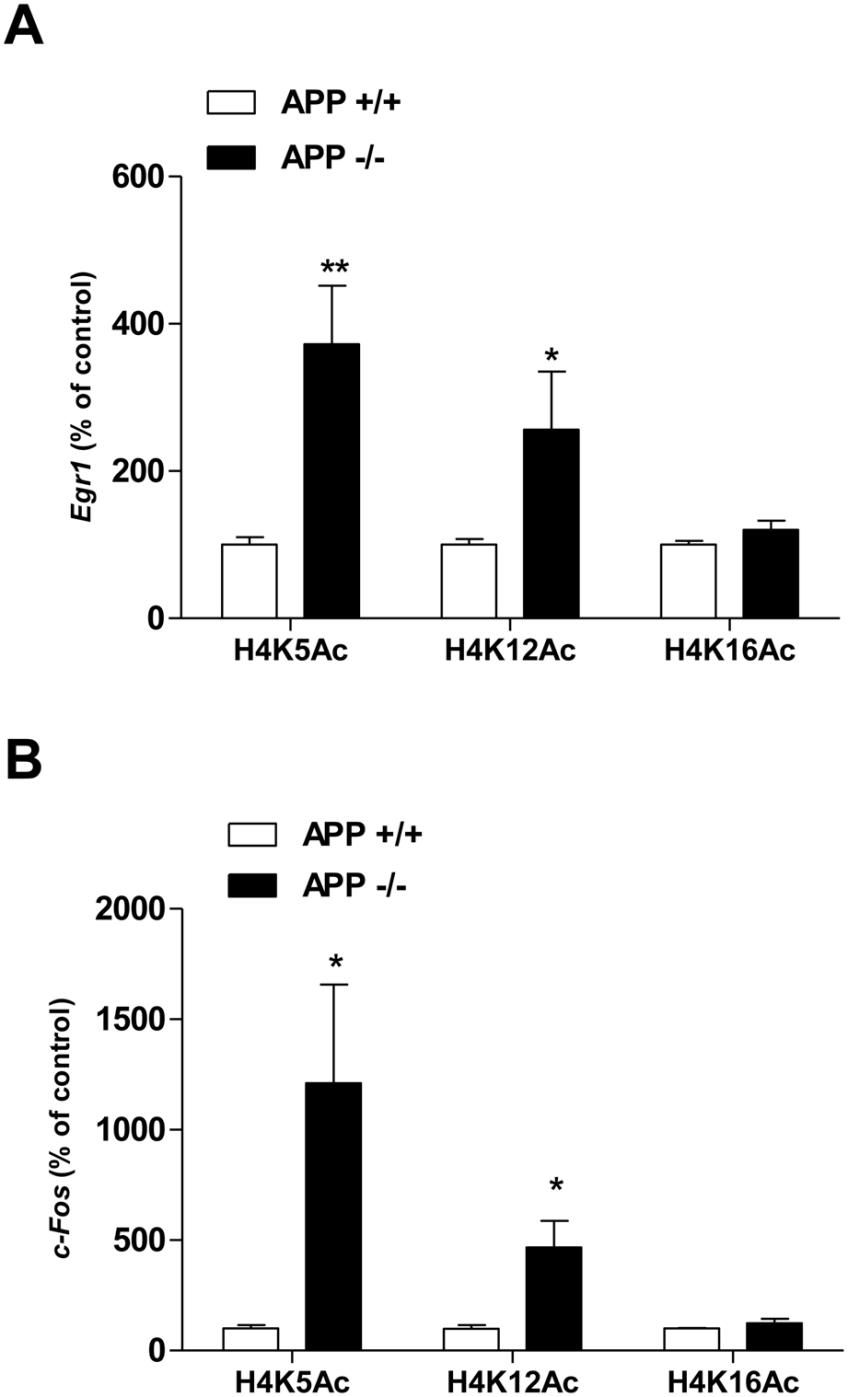
H4K5 and H4K12 are enriched at *Egr1* and *c-Fos* gene promoter in APP−/− mice. ChIP method was used to evaluate the enrichment of H4K12Ac, H4K5Ac, and H4K16Ac. A) *Egr1* promoter. B) *c-Fos* promoter. Data represent the level of enrichment normalized as percentage of APP+/+. ***p<0.001. All results derive from at least 3 mice per group and per antibody, and are expressed as mean ± SD.

Acetylation of histone H4 can occur at different positions including lysines 5, 12 and 16. To identify the positions that are acetylated on H4 enriched on the *Egr1* and *c-Fos* gene promoters in APP−/− mice, ChIP experiments were performed using anti-H4K5Ac, -H4K12Ac and -H4K16Ac specific antibodies. Results presented in figure 3A indicate that histones H4 enriched on the *Egr1* gene promoter in APP−/− mice were acetylated on positions K5 and K12, but not on K16. Interestingly, the same profile of acetylation was found for histone H4 enriched on the *c-Fos* gene promoter in APP−/− mice (Figure 3B). These results suggest that APP regulates *Egr1* and *c-Fos* gene transcription by similar molecular mechanisms.

### 4. Regulation of *Egr1* Gene Transcription by APP is CREB Dependent

**Figure 4:**
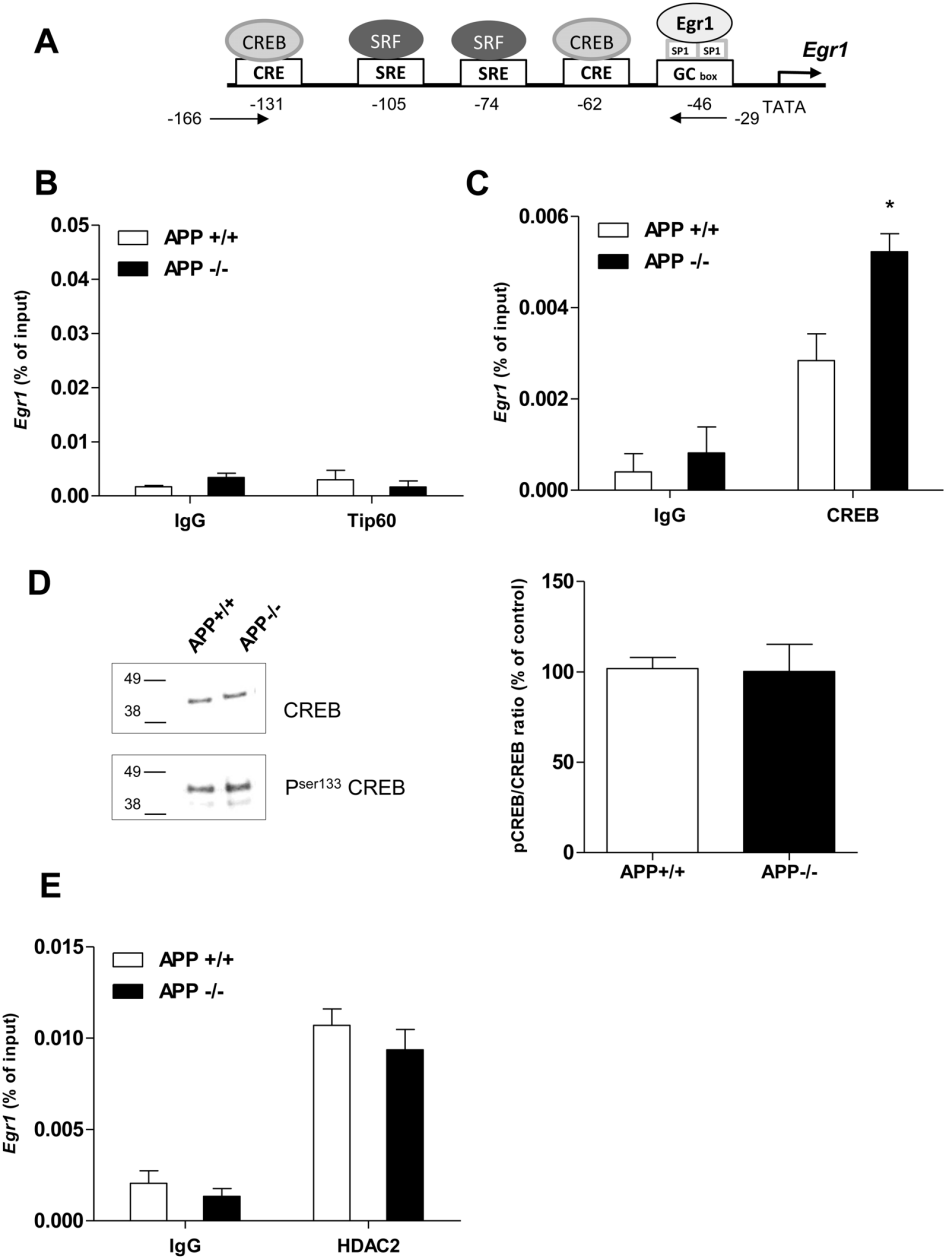
CREB is better recruited on the *Egr1* gene promoter in APP−/− mice. A) Schematic representation of the structure of *Egr1* gene promoter containing several binding sites for transcription factors and localization of primers utilized for q-PCR (CREB: cAMP response element, CRE: cAMP response elements SRF: Serum Response factor, SRE: Serum Response Element, SP1: specificity protein). B) Tip60 binding to *Egr1* gene promoter in APP+/+ and APP−/− PF cortex was assessed by ChIP using anti-Tip60 antibody. C) CREB binding to *Egr1* promoter in APP+/ and APP−/− mice. IgG was used as negative control. Equal amounts of ChIP and input DNA were used for qRT-PCR analysis on the *Egr1* gene promoter. Results show a significantly lower enrichment of CREB in APP+/+ mice, *p<0.05. Enrichment values were normalized to input values and represented the average of three or more experiments per group. Results are expressed as mean ± SD. D) Ratio between total and phosphorylated CREB was detected by western blot analysis of nuclear extracts from PF cortex of APP+/+ and APP−/− mice, 5 months of age (n = 5). Typical blot is shown, CREB/P-CREB ratio were quantified, and expressed as percentage of the APP+/+ mice. Data are expressed as mean ± SD. E) ChIP assay on Egr1 promoter using HDAC2 antibody.

A schematic representation of the *Egr1* gene promoter is given in figure 4A [22]. The histone acetyltransferase Tip60 is able to acetylate histone H4 at positions H4K5 and H4K12 [23] and interacts with AICD [24]. Therefore, an interaction between Tip60 and APP could inhibit H4K5 and H4K12 acetylation in APP+/+ mice. To test this hypothesis, ChIP experiments were performed using anti-Tip60 antibodies. Tip60 is able to bind to the promoter of *KAI1* gene and regulates its transcription [25], [26]. In Chip experiments, we indeed demonstrated an enrichment of Tip60 on the promoter of the *KAI1* gene (Figure S3). However, results presented in figure 4B do not show any enrichment of Tip60 on the *Egr1* gene promoter in APP−/− mice, allowing us to rule out the implication of Tip60 in this regulation. On the other hand, acetylation of H4K5 and H4K12 is mediated by CBP/P300, which associates with phosphorylated CREB DNA binding protein [27]. In addition, the *Egr1* promoter contains two CREB responsive elements (CRE) (Figure 4A). Therefore we attempted to immunoprecipitate CREB on *Egr1* promoter and we confirmed that this transcription factor was significantly more enriched on the *Egr1* gene promoter in APP−/− mice (figure 4C). To test whether APP was able to down regulate CREB expression or to inhibit its phosphorylation, nuclear extracts from APP+/+ and APP−/− mice were analyzed in Western blotting using anti-CREB and anti-S133PhosphoCREB specific antibodies. Results presented in figure 4D indicate the same CREB/PhosphoCREB ratio in APP+/+ and APP−/− mice, ruling out modification of CREB expression and phosphorylation by APP. The recruitment of CREB on a gene promoter is often followed by local depletion of several HDACs [28], [29]. As HDAC2 epigenetically regulates transcription of several IEGs including *Egr1* [30], we also performed ChIP experiments using HDAC2 antibodies. Results presented in figure 4E do not show any significant enrichment of HDAC2 on the *Egr1* gene promoter in APP−/− mice. Altogether, these results indicate that APP fosters low level of *Egr1* expression in mouse brain by inhibiting recruitment of CREB without affecting enrichment of HDAC2 on the *Egr1* gene promoter.

### 5. Regulation of *c-Fos* Gene Transcription by APP is HDAC2 Dependent

**Figure 5:**
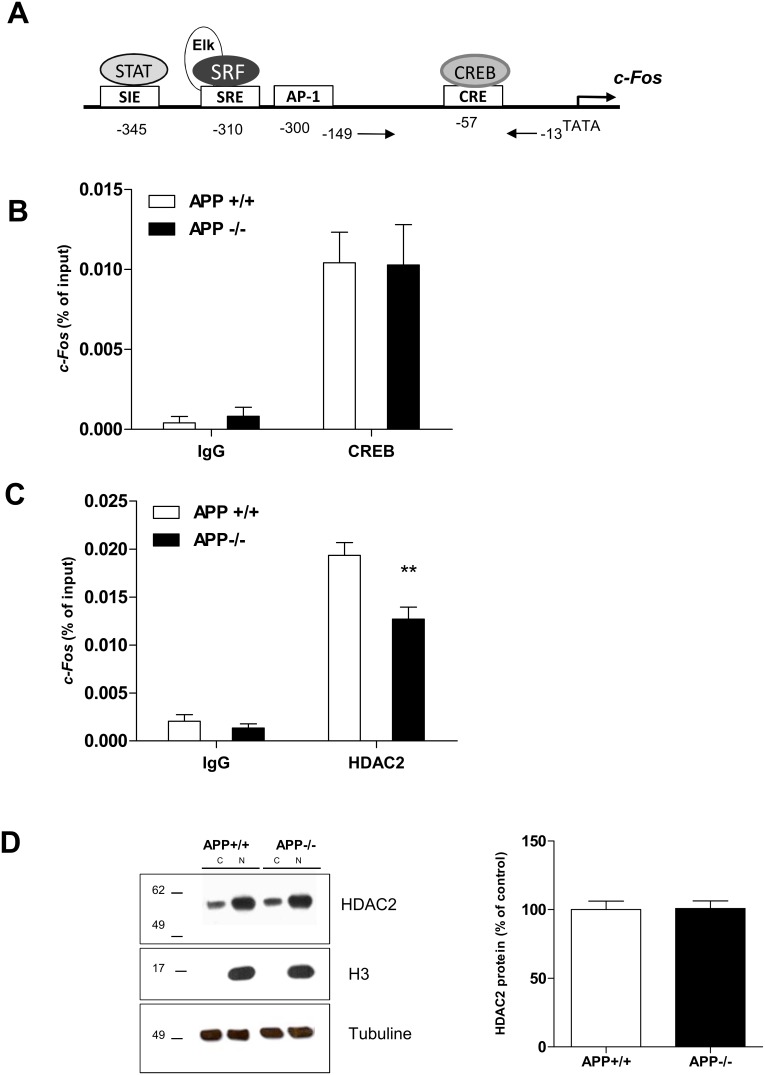
HDAC2 is enriched on the *c-Fos* gene promoter in APP−/− mice. A) Schematic representation of the structure of *c-Fos* promoter containing only one CREB binding site and the localization of the primers utilized for q-PCR. (CREB: cAMP response element, CRE: cAMP response elements SRF: Serum Response factor, SRE: Serum Response Element, AP1: Activator protein 1, SIE: sis-inducible element, STAT: Signal Transducer and Activator of Transcription). B) Study of CREB binding to c-Fos gene promoter in APP+/and APP−/− mice. C) ChIP assay on *c-Fos* gene promoter using anti-HDAC2 antibody. Results show a significant lower enrichment of HDAC 2 in PF cortex of APP−/− mouse, Student’s t-test: **p<0.01. IgG was used as negative control. Equal amounts of ChIP and input chromatin were used for qRT-PCR analysis on the *c-Fos* gene promoter. Enrichment values were normalized to input values and represented the average of three or more mice per experiment. Results are expressed as mean ± SD. D) HDAC2 protein expression was detected by western blot analysis of nuclear (N) or cytoplasmic (C) extracts obtained from PF cortex of APP+/+ and APP−/− mice, 5 months of age (n = 5). Typical blot is shown with anti-histone H3 and anti-tubulin used as a loading control. Data are normalized against histone H3 to assess the level of nuclear HDAC2 and expressed as mean ± SD.

The *c-Fos* gene promoter also contains a single CRE site (Figure 5A). The same profile of acetylation of histone H4 enriched on *c-Fos* and *Egr1* gene promoters in APP−/− mice (Figure 3B) suggested that APP might regulate *Egr1* and *c-Fos* gene transcription by similar molecular mechanisms. Therefore, we first measured recruitment of CREB on the *c-Fos* gene promoter in APP+/+ and APP−/− mice. Results of ChIP experiments (Figure 5B) show an enrichment of CREB on *c-Fos* promoter, but no difference between APP+/+ and APP−/− mice, suggesting that basal regulation of *c-Fos* gene transcription by APP is not CREB dependent although c-Fos is regulated by CREB [31]. Since HDAC2 was previously demonstrated to inhibit *c-Fos* gene transcription [30], we performed ChIP experiments using anti-HDAC2 antibodies. Results presented in figure 5C clearly indicate a significant enrichment of HDAC2 on the *c-Fos* gene promoter in APP+/+ mice. Western blot analysis of nuclear extracts prepared from the PF cortex of APP+/+ and APP−/− mice with anti-HDAC2 antibodies did not show any modification of HDAC2 nuclear or cytoplasmic content, indicating that APP does not modify neither HDAC2 expression nor its nuclear localization (Figure 5D). Altogether, these results indicate that APP fosters a low level of *c-Fos* expression in mouse brain by increasing enrichment of HDAC2 on the *c-Fos* gene promoter.

### 6. Exposure of Mice to Novelty Induces IEGs Transcription in APP+/+ but not in APP−/− Mice

**Figure 6:**
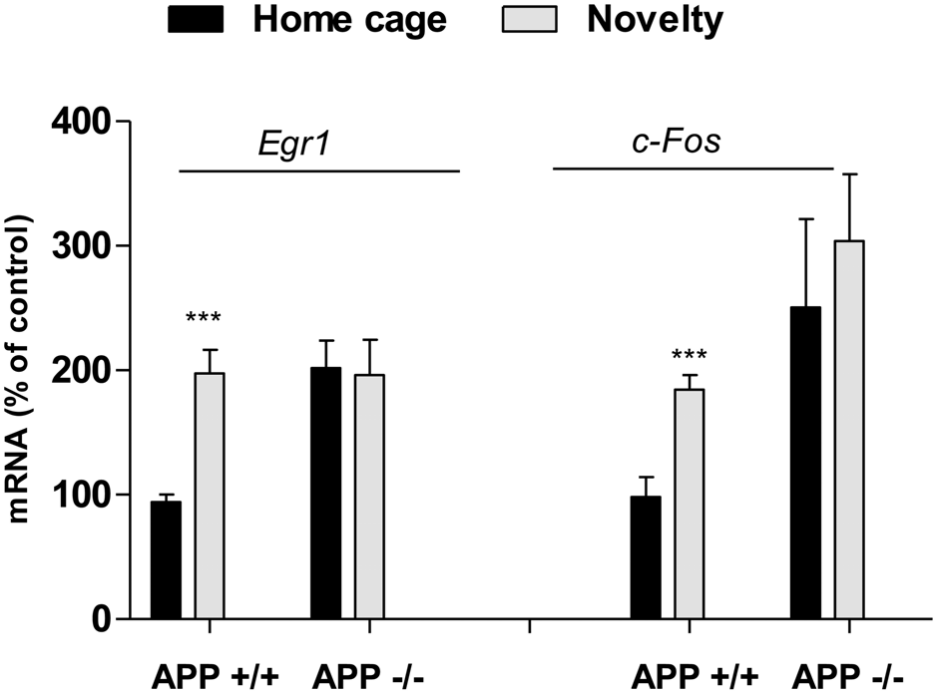
APP+/+ but not APP−/− mice induce *Egr1* and *c-Fos* expression after exposure to novelty. A) Effect of exposure to novelty on the levels of *Egr1* and *c-Fos* transcripts. qRT PCR were performed on mRNA extracted from APP+/+ and APP−/− mice exposed (Novelty) or not (Home cage) to the open field (n = 6 or more per group). All values were normalized to the GAPDH mRNA, and expressed as percentage of the APP+/+. Results are expressed as mean ± SD. Student’s t-test. (***p<0.001).

*Egr1* and *c-Fos* are inducible transcription factors that are needed for synaptic plasticity and establishment of long term memory. We reasoned that induction of IEGs transcription needs a constitutive low level of expression, as measured for *Egr1* and *c-Fos* in APP+/+ mice. APP+/+ and APP−/− mice were exposed to novelty during a spatial exploration of an open field, in an experimental protocol that was previously described to enhance *c-Fos* and *Egr1* mRNA levels in mouse PF cortex [10], [32]. After a short period of exploration of 15 min and a resting period of 30 min, the mRNA levels of IEGs were quantified in APP+/+ and APP−/− mice. Results presented in figure 6 clearly demonstrated that induction of both *Egr1* and *c-Fos* gene transcription was possible in APP+/+ but not in APP−/− mice. These results suggest that the low level of IEGs transcripts measured in APP+/+ allow their induction during exposure of mice to novelty, while such an induction is not possible in APP−/− mice showing a high basal level of IEGs expression.

## Discussion

The main finding of this study is that APP fosters low expression of *c-Fos* and *Egr1* in mouse PF cortex, in which induction of several IEGs is involved in memory formation [33]–[35]. APP-mediated down regulation of *c-Fos* and *Egr1* by two distinct epigenetic mechanisms is needed to induce properly transcription of these IEGs upon exposure of mice to novelty.

We extended our observation of the epigenetic regulation of *Egr1* gene transcription by APP to *c-Fos*, *Bdnf* and *Arc*. All of those genes belong to the IEGs family of transcription or neurotrophic factors needed for memory formation [2], [7], [36], [37]. The ability of APP to increase gene transcription by an AICD dependent mechanism has been previously demonstrated, although still debated [38]–[43]. Here, we show for the first time that APP represses transcription of a group of genes, all related to synaptic plasticity. The moderate effect of APP on IEGs expression that we measured could explain why these IEGs have never been reported as APP target genes in microarray experiments in which the cut off of differences in gene expression is usually higher.

Induction of IEGs through acetylation of histones H3, H4 and H2B is well documented [44], and acetylation of histone H3K14, H4K12 or phosphorylation of H3S10 via ERK activation are associated with chromatin relaxation leading to IEGs transcription and memory formation [45]–[47]. We have previously demonstrated that trichostatin A, a specific HDAC inhibitor, was able to induce *Egr1* gene transcription in both APP+/+ and APP−/− neurons, although induction was significantly higher in APP−/− neurons, in agreement with higher H4 acetylation at the *Egr1* gene promoter in these cells [20].

Although induction of IEGs has been widely studied, little is known about basal regulation of IEGs transcription in the hippocampus and the cortex. Basal expression of IEGs in the brain is referred as an expression induced by physiological synaptic input [48]. We demonstrate here that APP fosters a low level of expression of the four IEGs studied. APP represses transcription of *Egr1*, *c-Fos*, and *Bdnf* by decreasing enrichment of acetylated H4, but not H3 nor H2B histones on the corresponding gene promoters. APP-mediated repression of *Arc* gene transcription appears to be controlled by other molecular mechanisms. SAHA, a nonspecific HDAC inhibitor, was demonstrated to up regulate c-Fos and Bdnf expression, while Arc induction was much more moderate [49], indicating that Arc expression should be regulated by other mechanisms than histone modification.

We observed a very similar acetylation profile of H4K5 and H4K12, but not H4K16, on *Egr1* and *c-Fos* gene promoters, which suggested that APP might regulate both genes by similar mechanisms. H4K5 and H4K12 are known to be acetylated by Tip60 and CBP/P300 histone acetyltransferase activities [23]. As Tip60 interacts with AICD and the adaptor protein Fe65 [24], APP could inhibit H4K5 and H4K12 acetylation in APP+/+ mice. We analyzed a possible interaction of Tip60 with *Egr1* promoter but failed to detect any significant interaction or any enrichment in APP−/− mice. Therefore, the contribution of CBP/P300 in the acetylation of H4K5 and H4K12 present on the *Egr1* and *c-Fos* gene promoters was further investigated.

CBP and p300 are two highly related histone acetyltransferases that share many biological functions and interact with phosphorylated cAMP response element binding protein (CREB) [50]. CRE sites are present on *Egr1* and *c-Fos* gene promoters, and regulation of *Egr1* and *c-Fos* gene transcription by CREB is well established [19], [21], [31], [51]. We demonstrate that regulation of *Egr1* gene transcription by APP is CREB dependent. In APP−/− mice, CREB is better recruited on the *Egr1* gene promoter, arguing for a CBP/P300-mediated increase in H4K5 and H4K12 acetylation of H4 present on the *Egr1* promoter. The presence of CREB on a gene promoter has been associated with the removal of HDACs [28]. HDAC2 deacetylates H4K5 and H4K12, and is described as a negative actor in memory formation and synaptic plasticity [30]. Even if we were able to detect enrichment of HDAC2 on *Egr1* gene promoter, the level of HDAC2 detected was the same in APP+/+ and APP−/− mice.

Surprisingly, regulation of *c-Fos* gene transcription by APP occurs by a different mechanism, since the same recruitment of CREB on the *c-Fos* gene promoter was measured in both APP+/+ and APP−/− mice but less HDAC2 was found on the *c-Fos* gene promoter in APP−/− mice. APP decreased CREB recruitment on *Egr1* gene promoter and increased HDAC2 recruitment on *c-Fos* gene promoter, without any modification of either CREB and HDAC2 expression or CREB phosphorylation. The presence of two CRE sites in the *Egr1* gene promoter could explain why APP-mediated regulation of this gene is more dependent on CREB recruitment.

Our results indicate that APP fosters a low expression of a group of IEGs involved in memory formation. Consequently, overexpression of APP could have important consequences on memory formation. Interestingly, it was previously demonstrated that in APP transgenic mice, overexpression of mutated APP decreases the basal levels of IEGs mRNA and impairs the proper induction of IEGs transcription upon exposure to novelty [32]. Similarities in the cognitive declines observed in both APP−/− and APP transgenic mice [52]–[54] could be related to their impairment in inducing properly IEGs transcription. Moreover, impairment of LTP observed in APP transgenic mice have been associated with dysregulation of histone H4 acetylation [55], [56].

Basal expression of IEGs is important for normal synaptic activity, but their rapid induction is needed to activate transcription of many genes playing a key role in establishment of long term memory [7]. An important function of APP is to epigenetically foster low level of expression of IEGs, allowing rapid induction of their transcription.

## Supporting Information

Figure S1 **Housekeeping genes and other IEGs expressions are not regulated by APP.** *GAPDH* mRNA levels were quantified by q-RT PCR in APP+/+ and APP−/− PF cortex (n = 6) and normalized versus Actin B) q-RT PCR method was used to assess mRNA levels of the housekeeping genes *Actin, peptidylprolyl isomerase A (Ppia)* and *β-glucuronidase (Gusb)* in APP+/+ and APP−/− PF cortex (n = 6). C) mRNA levels of the IEGs *c-Jun* and *Homer-1a* were quantified by q-RT PCR in in APP+/+ and APP−/− PF cortex (n = 6). Values were normalized to the GAPDH mRNA, and expressed as percentage of APP+/+, mean ± SD. Primers sequences used were as follows: Ppia FOR: CAGACGCCACTGTCGCTTT; Ppia REV: TGTCTTTGGAACTTTGTCTGCAA, Gusb FOR: ACTGACACCTCCATGTATCCCAAG, Gusb REV: CAGTAGGTCACCAGCCCGATG, c-Jun FOR: TGAAAGCTGTGTCCCCTGTC; c-Jun REV: ATCACAGCACATGCCACTTC, Homer 1a FOR: GAAGTCGCAGGAGAAGATG, Homer1a REV: TGATTGCTGAATTGAATGTGTACC.
(TIF)

Figure S2 **Analysis of histone acetylation by ChIP assays on GAPDH promoters.** ChIP experiments were performed on chromatin obtained from APP+/+ and APP−/− PF cortex. Immunoprecipitation was completed with antibody recognizing normal mouse IgG as negative control, anti H3Ac, H2BAc and H4Ac. The quantification of immunoprecipitated DNA and the normalization versus total DNA (input) was assessed by real-time qPCR with primers designed on GAPDH. All results were obtained from at least 3 or more mice per group and per antibody, and are expressed as mean ± SD.
(TIF)

Figure S3 **Tip60 is enriched in KAI1 promoter.** Tip60 binding to *KAI1* gene promoter in APP+/+ and APP−/− PF cortex was assessed by ChIP using anti-Tip60 antibody, with primers designed in KAI1 promoter region. Primers sequences used were as follows: KAI1 FOR: ACCGTTAGGCAGCGCCGTGAG; KAI1 Rev: CTTGGGAAGGCGGTGCGCTC. IgG was used as negative control. Results show a significant enrichment of Tip60 in APP+/+ mice, **p<0.01. Enrichment values were normalized to input values and are the average of three or more experiments per group. Results are expressed as mean ± SD.
(TIF)
