# Supplementary figures and images for: Epigenetic Regulations of Immediate Early Genes Expression Involved in Memory Formation by the Amyloid Precursor Protein of Alzheimer Disease
Source: PLoS One. 2014 Jun 11;9(6):e99467. doi: 10.1371/journal.pone.0099467 (PMC4053420; doi:10.1371/journal.pone.0099467)

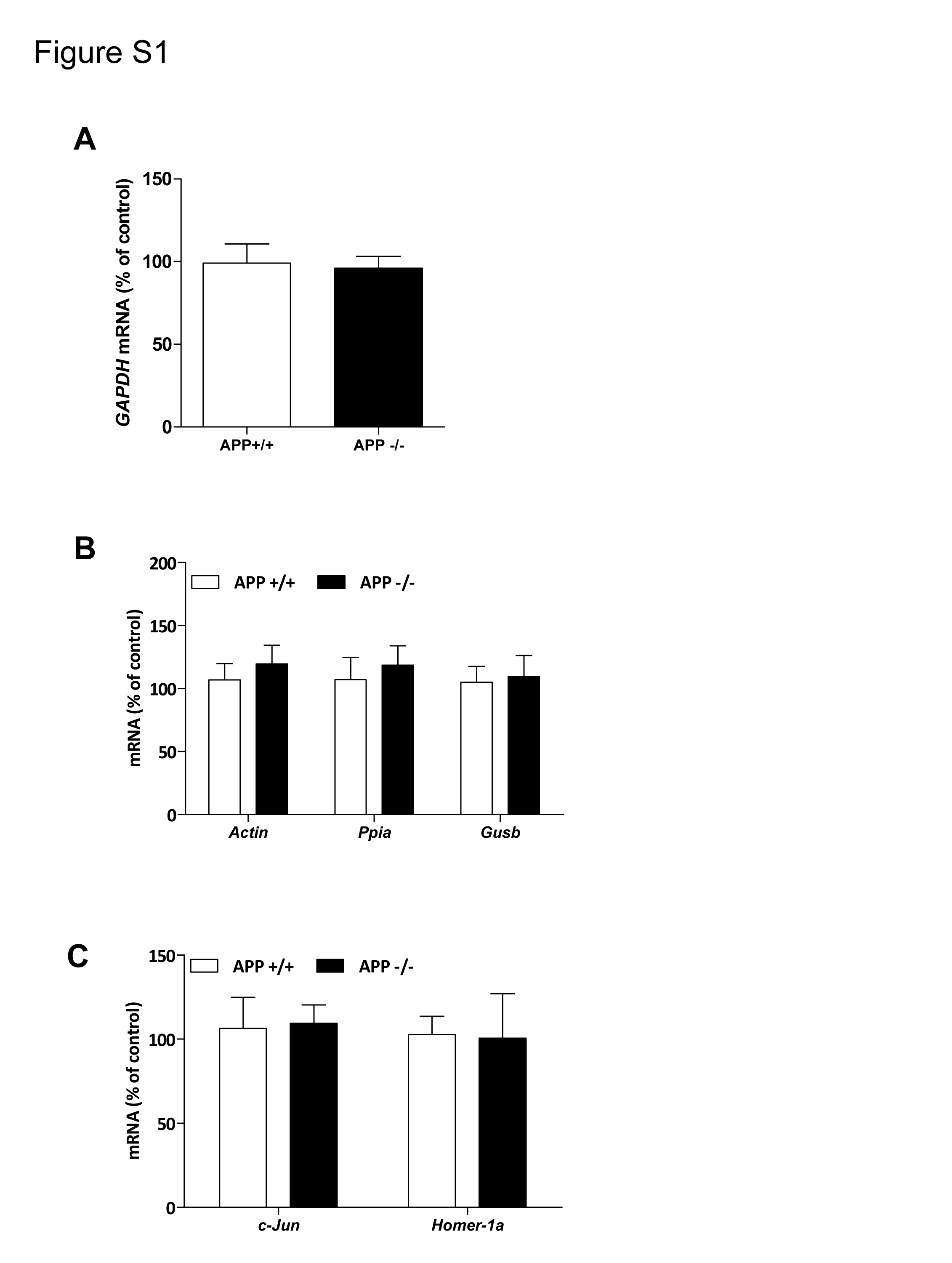

Supplement: Figure S1 — Housekeeping genes and other IEGs expressions are not regulated by APP. GAPDH mRNA levels were quantified by q-RT PCR in APP+/+ and APP−/− PF cortex (n = 6) and normalized versus Actin B) q-RT PCR method was used to assess mRNA levels of the housekeeping genes Actin, peptidylprolyl isomerase A (Ppia) and β-glucuronidase (Gusb) in APP+/+ and APP−/− PF cortex (n = 6). C) mRNA levels of the IEGs c-Jun and Homer-1a were quantified by q-RT PCR in in APP+/+ and APP−/− PF cortex (n = 6). Values were normalized to the GAPDH mRNA, and expressed as percentage of APP+/+, mean ± SD. Primers sequences used were as follows: Ppia FOR: CAGACGCCACTGTCGCTTT; Ppia REV: TGTCTTTGGAACTTTGTCTGCAA, Gusb FOR: ACTGACACCTCCATGTATCCCAAG, Gusb REV: CAGTAGGTCACCAGCCCGATG, c-Jun FOR: TGAAAGCTGTGTCCCCTGTC; c-Jun REV: ATCACAGCACATGCCACTTC, Homer 1a FOR: GAAGTCGCAGGAGAAGATG, Homer1a REV: TGATTGCTGAATTGAATGTGTACC. (TIF) [file pone.0099467.s001.tif]

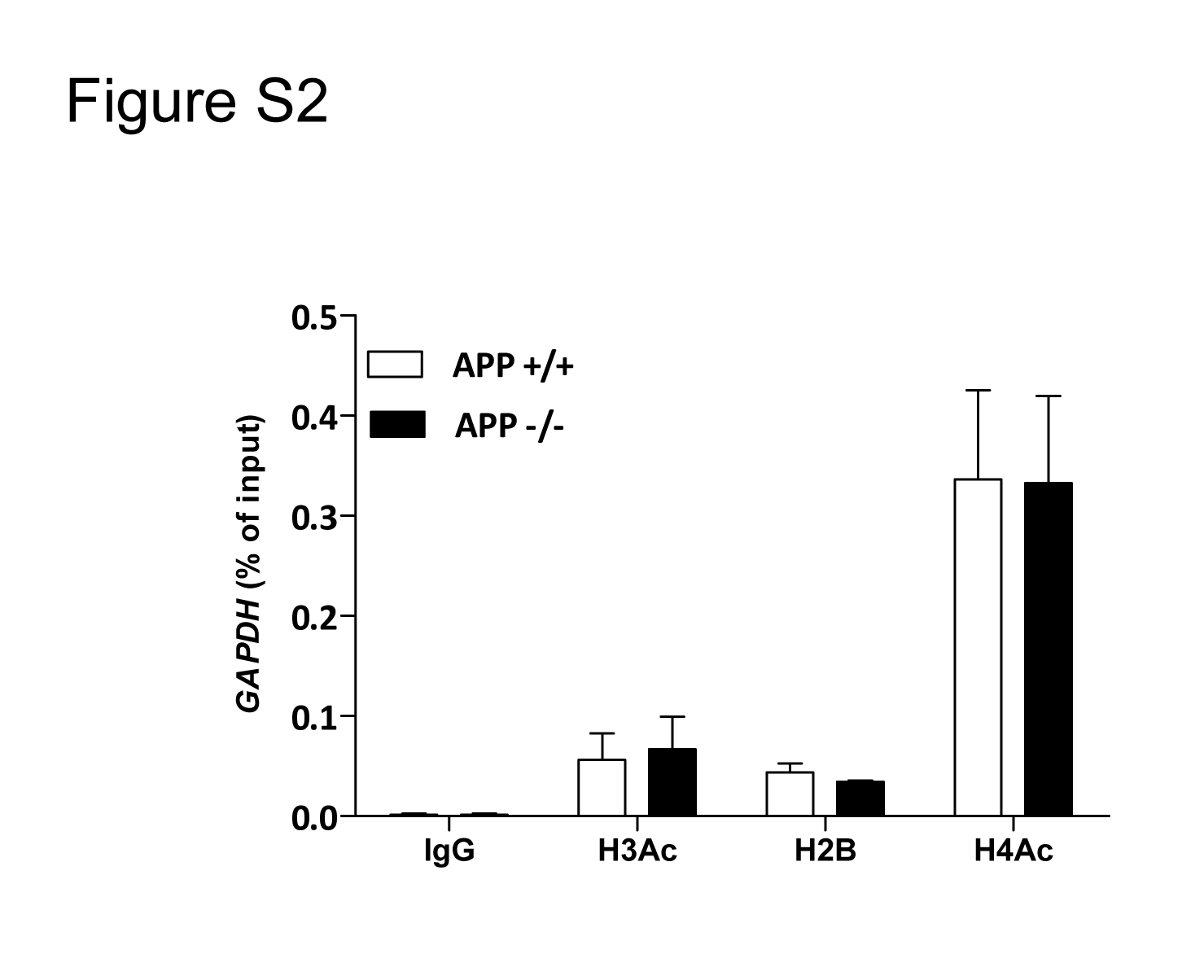

Supplement: Figure S2 — Analysis of histone acetylation by ChIP assays on GAPDH promoters. ChIP experiments were performed on chromatin obtained from APP+/+ and APP−/− PF cortex. Immunoprecipitation was completed with antibody recognizing normal mouse IgG as negative control, anti H3Ac, H2BAc and H4Ac. The quantification of immunoprecipitated DNA and the normalization versus total DNA (input) was assessed by real-time qPCR with primers designed on GAPDH. All results were obtained from at least 3 or more mice per group and per antibody, and are expressed as mean ± SD. (TIF) [file pone.0099467.s002.tif]

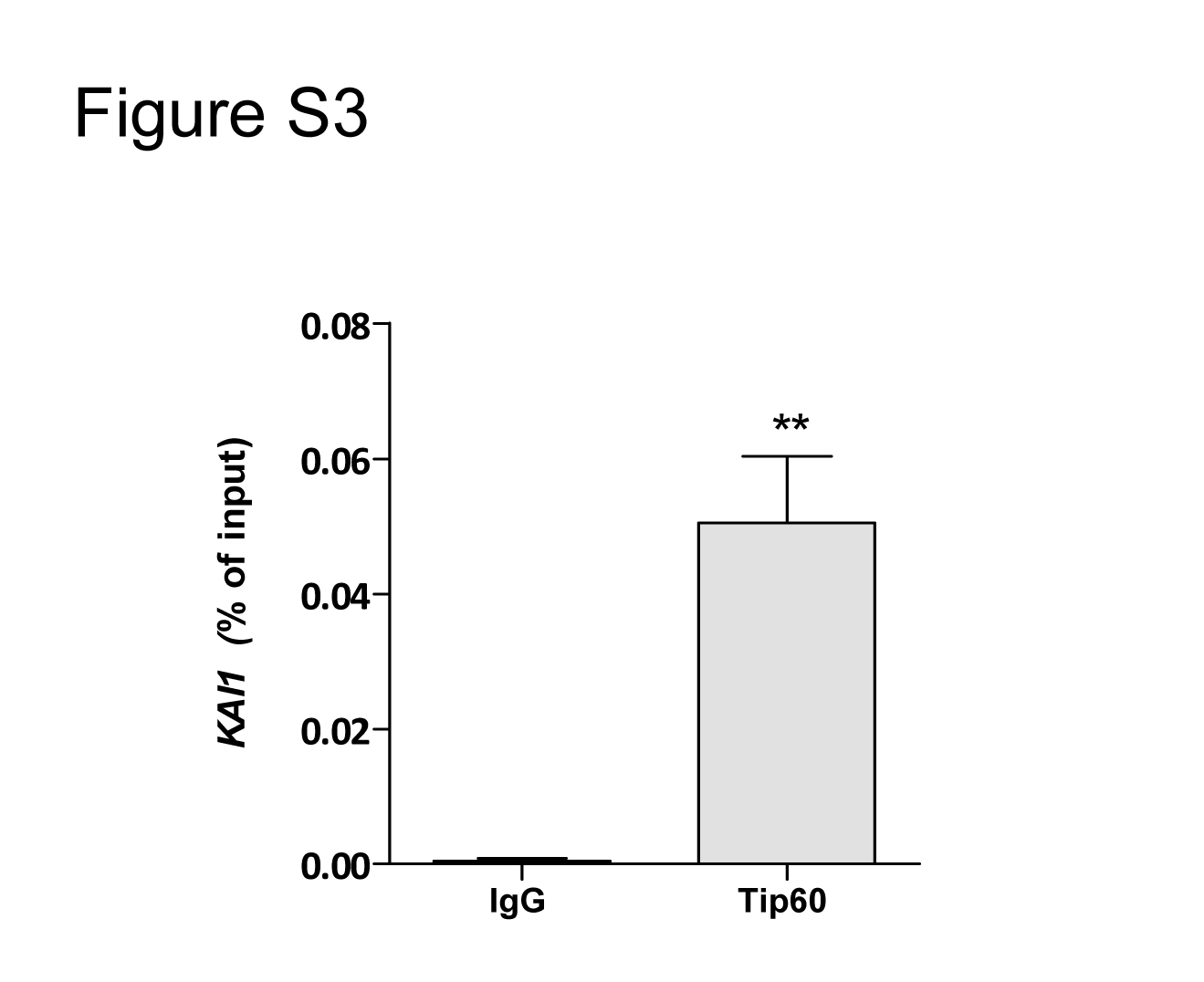

Supplement: Figure S3 — Tip60 is enriched in KAI1 promoter. Tip60 binding to KAI1 gene promoter in APP+/+ and APP−/− PF cortex was assessed by ChIP using anti-Tip60 antibody, with primers designed in KAI1 promoter region. Primers sequences used were as follows: KAI1 FOR: ACCGTTAGGCAGCGCCGTGAG; KAI1 Rev: CTTGGGAAGGCGGTGCGCTC. IgG was used as negative control. Results show a significant enrichment of Tip60 in APP+/+ mice, **p<0.01. Enrichment values were normalized to input values and are the average of three or more experiments per group. Results are expressed as mean ± SD. (TIF) [file pone.0099467.s003.tif]
